# Twelve-week-conjugated linoleic acid supplementation has no effects on the selected markers of atherosclerosis in obese and overweight women

**DOI:** 10.3402/fnr.v60.32776

**Published:** 2016-11-08

**Authors:** Monika Dus-Zuchowska, Edyta Madry, Patrycja Krzyzanowska, Paweł Bogdanski, Jaroslaw Walkowiak

**Affiliations:** 1 Department of Pediatric Gastroenterology and Metabolic Diseases, Poznan University of Medical Sciences, Poznan, Poland; 2 Department of Physiology, Poznan University of Medical Sciences, Poznan, Poland; 3 Department of Education and Obesity Treatment and Metabolic Disorders, Poznan University of Medical Sciences, Poznan, Poland

**Keywords:** conjugated linoleic acid, atherosclerosis, high-sensitivity C-reactive protein, asymmetrical dimethylarginine

## Abstract

**Background:**

The antiatherogenic effect of conjugated linoleic acid (CLA) has been demonstrated in animal models. Although there are plenty of *in vitro* studies that suggest the profitable properties of CLA, the results in humans remain inconsistent.

**Objective:**

In this study, we assessed the impact of CLA supplementation on the levels of atherosclerosis markers – high-sensitivity C-reactive protein (hs-CRP) and asymmetrical dimethylarginine (ADMA).

**Design:**

Seventy-four adult female subjects with body mass index ≥25 kg/m^2^ were enrolled in the double-blind, placebo-controlled nutritional intervention. The study participants were randomly assigned to receive 3 g/day CLA or placebo (sunflower oil) for 12 weeks. In all subjects, we measured hs-CRP and ADMA concentrations by using enzyme-linked immunosorbent assay.

**Results:**

No significant differences were found in hs-CRP and ADMA levels before and after nutritional intervention between both groups. The changes in hs-CRP and ADMA concentration values (Δhs-CRP; ΔADMA median [interquartile range]) did not differ between subjects from the placebo (−0.1 [−0.8 to 0.3]; −0.02 [−0.12 to 0.14]) and CLA (0.2 [−0.7 to 0.9]; 0.04 [−0.14 to 0.13]) groups. The incidence of reduction of hs-CRP or ADMA concentration was not different in subjects of the CLA group compared to those of the placebo group (41.9% vs. 50%, relative risk [RR]=0.8387, 95% confidence interval [CI]=0.4887–1.4493, *p*=0.5232 and 61.3% vs. 56.2%, RR=1.0896, 95% CI=0.7200–1.6589, *p*=0.6847, respectively).

**Conclusion:**

Twelve weeks of CLA supplementation had no effect on selected markers of atherosclerosis in obese and overweight women.

Increased interest in functional foods has led to an improved understanding of their putative health benefits. In the omnipresent medication-based treatment of human problems and the inevitable side effects, natural products such as conjugated linoleic acid (CLA) could be a favorable, safe supplement therapy (1). CLA is a group of isomers of octadecadienoic acids, naturally produced in the gastrointestinal system of ruminants through microbial biotransformation of forage-derived fatty acids. The endogenous production of CLA in humans is possible from dietary-derived vaccenic acid, but CLA concentrations in human blood and tissues are low (2). The major sources of CLA for humans are dairy products and ruminant meats such as lamb and beef. There are also commercially available forms of CLA obtained through alkaline isomerization of linoleic acid–rich oils (3). The potential health benefits of CLA supplementation studied on animal models since 1987 include body fat reduction, improved insulin resistance, improved lipid profile, modulation of the immune system, stimulation of bone mineralization, anticarcinogenic effects, and prevention of atherogenesis (2, 4, 5). Bruen et al. (6) suggested the antiatherogenic effect of CLA is due to inhibition of the inflammatory response to the damage of the endothelium and modulation of circulating cholesterol. In animal models, the regression of the atherosclerotic lesions was associated with downregulation of proinflammatory genes and induction of apoptotic processes (7).

The advantages of CLA supplementation, shown in animal models, seem very attractive to the human population, especially with regard to its potential antiatherogenic effect. Since the last century, atherosclerosis (AT), with its wide spectrum of clinical manifestations, particularly ischemic heart disease and stroke, ranks among the top causes of death in the Western population. Obesity is one of the strongest risk factors of AT (8). According to World Health Organization data, in 2014 nearly two billion adults worldwide were overweight (39% of men and 40% of women), and, of these adults, more than half a billion were obese (11% of men and 15% of women) (9). These data obligates scientists to broaden the knowledge about possible new antiatherogenic and antiobesity agents such as CLA.

In this study, we assessed the impact of 12 weeks of supplementation of CLA on the levels of selected AT markers – high-sensitivity C-reactive protein (hs-CRP) and asymmetrical dimethylarginine (ADMA) in overweight and obese women.

## Methods

### Participant characteristics

Seventy-four female subjects aged over 18 years were enrolled in the study. Participants were recruited from the Obesity and Overweight Treatment Clinic of Poznan University of Medical Sciences, Poznan, Poland. Inclusion criteria were defined as follows: female sex, age over 18 years, and body mass index (BMI) ≥25 kg/m^2^. Exclusion criteria included a history of chronic systemic disease (with the exception of hypertension [HT]); celiac disease; type 2 diabetes; pancreatic or liver disease, or both; current or recent (within the preceding month) treatment with CLA or agents interfering with fat digestion or absorption, or both (e.g. chitosan, orlistat, green tea); and pregnancy (10).

**Fig. 1:**
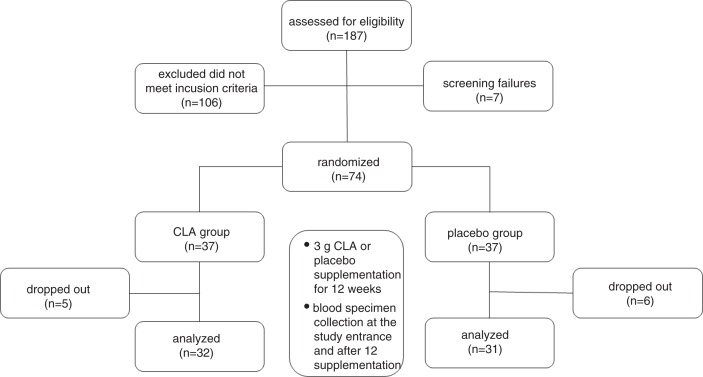
Flowchart illustrating the progression of participants through the study.

The flowchart illustrating the progression of participants through the study is presented in Fig. 1 (10, 11). Of the 187 registered women, 81 met the inclusion criteria of the study. Seven of those participants were excluded because of screening failures (shortage of time – 3; diarrhea, stomach pain – 1; difficulty in cooperation – 1; personal problems – 1; suspicion of ovarian cancer – 1). The randomization process included 74 participants. Thirty-seven of them formed the experimental CLA group and the rest were assigned to the placebo group. The dropout rate in the CLA group was comparable to the placebo group (13.51% vs. 16.22%). The dropouts included people who 1) missed the final study visit (3 women from the CLA group and 4 from the placebo group), 2) reported side effects (e.g. nausea) of the tested product (1 woman from the CLA group and 2 from the placebo group), and 3) became pregnant (1 woman from the CLA group).

A physical examination that included an evaluation of body weight, height, and BMI of each participant was conducted upon entry to the study. Baseline characteristics of the study group are summarized in Table 1.

**Table 1:** Baseline characteristics of the study group

	CLA group (*n*=37)		Placebo group (*n*=37)		
			
	Median	First–third quartile	Median	First–third quartile	*p*
Age (years)	54	43–59	54	45–61	0.9914
BMI (kg/m^2^)	34.00	30.07–37.58	35.36	31.75–38.62	0.5049
CRP (mg/L)	2.7	1.4–3.6	2.9	1.5–4.0	0.5688
ADMA (nmol/mL)	1.55	0.58–2.62	1.42	0.65–2.37	0.9914

CLA, conjugated linoleic acid; BMI, body mass index; CRP, C-reactive protein; ADMA; asymmetrical dimethylarginine.

### Study design

This study was a randomized, double-blind, placebo-controlled nutrition trial. The study participants were randomly assigned to receive CLA or placebo (Olimp Laboratories, Debica, Poland). CLA and placebo products were identical (i.e. transparent capsules with a yellowish tint, packed in similar blisters). The CLA capsules contained 0.5 g of 80% CLA (50:50 *cis*-9, *trans*-11 and *trans*-10, *cis*-12 isomers), and placebo capsules contained 0.5 g of sunflower oil. The fatty acid compositions of both capsules were presented in our previous article (11).

During the randomization process, a computer-generated number was assigned to each study participant. The randomization codes were hidden from investigators and participants until the end of all data analyses.

All participants enrolled in the study were instructed to administer two capsules of the provided substance three times a day (3 g of CLA or placebo) with a meal for 12 weeks. The compliance was checked by 1) a monthly phone call to every participant by a member of the research group and 2) participants marking pill administration in a special calendar. The completion criterion was consumption of 75% of the supplement provided. The participants were asked to maintain their current lifestyle, including current physical activity and eating habits, during the trial period.

### Biochemical analysis

A venous blood sample was collected, according to standardized methods, following an overnight fast and was immediately centrifuged. The plasma was poured into a tube and stored at −70°C until analyzed. In all study participants, hs-CRP and ADMA levels were measured by enzyme-linked immunosorbent assay (human ADMA, SunRed Biological Technology, Shanghai, China; hs-CRP, DRG International Inc., Springfield Township, New Jersey, USA).

### Statistics

Results are presented as means±standard errors of the mean and medians with interquartile ranges. Statistical analysis was carried out with STATISTICA 12 software (StatSoft Inc., Tulsa, OK, USA). The statistical significance of differences in hs-CRP and ADMA levels between the CLA and placebo groups was determined with Mann-Whitney *U* tests. The Wilcoxon-rank test was used for within-group analyses (baseline vs. 12 weeks). The significance level was set at *p<*0.05. Assuming a potential drop-out rate of 20%, a significance level of 5%, and the detection power of 80%, the sample size was calculated as 37 for each group (10, 11).

This study was conducted according to Declaration of Helsinki guidelines and all procedures involving human subjects were approved by the local Bioethics Committee of the Institutional Review Board at the Poznan University of Medical Sciences, Poland (approval 358/14). Written, informed consent was obtained from all participants. The trial was registered in German Clinical Trials Register (DRKS-ID: DRKS00010469; www.germanctr.de/)

## Results

### hs-CRP

Before and after nutrition intervention, there were no significant differences in hs-CRP levels between both groups (Table 2). The changes in hs-CRP concentration (Δhs-CRP) did not differ between subjects from the placebo and CLA groups (Table 3). The incidence of reduction of hs-CRP concentration was not different in subjects in the CLA group compared with those in the placebo group (41.9% vs. 50%, relative risk [RR]=0.8387, 95% confidence interval [CI]=0.4887–1.4493, *p*=0.5232).

**Table 2:** Values of hs-CRP (mg/L) for CLA and placebo groups before and after the 12-week nutritional intervention

	CLA (*n=*31)				Placebo (*n*=32)				
			
	Median	First–third quartile	Mean	SEM	Median	First–third quartile	Mean	SEM	*p*
Before	2.7	1.3–3.7	3.1	0.5	3.1	1.3–4.0	3.5	0.5	0.5688
After	2.1	1.5–4.5	3.2	0.5	2.6	0.8–4.2	3.3	0.6	0.6375
*p*	0.6733				0.4537				

hs-CRP, high-sensitivity C-reactive protein; CLA, conjugated linoleic acid; SEM, standard error of the mean.

**Table 3:** Values of Δhs-CRP in the CLA and placebo groups

	Δhs-CRP		
	
	CLA	Placebo	*p*
Median	0.2	−0.1	0.4160
First–third quartile	−0.7 to −0.9	−0.8 to −0.3	
Mean	0	−0.2	
SEM	0.3	0.4	

Δhs-CRP, change in high-sensitivity C-reactive protein; CLA, conjugated linoleic acid; SEM, standard error of the mean.

### ADMA

ADMA concentrations before and after supplementation in the CLA and placebo groups did not differ (Table 4). The changes in ADMA levels (ΔADMA) were not different in the groups studied (Table 5). The incidence of ADMA reduction was not different between the CLA group and the placebo group (61.3% vs. 56.2%, RR=1.0896, 95% CI=0.7200–1.6589, *p*=0.6847).

**Table 4:** Values of ADMA (nmol/mL) for the CLA and placebo groups before and after 12-week nutritional intervention

	CLA (*n=*31)				Placebo (*n=*32)				
			
	Median	First–third quartile	Mean	SEM	Median	First–third quartile	Mean	SEM	*p*
Before	1.58	0.56–2.75	1.65	0.19	1.36	0.59–2.29	1.57	0.19	0.9914
After	1.33	0.59–2.79	1.66	0.20	1.13	0.57–2.40	1.55	0.19	0.6771
*p*	0.7688				0.5632				

ADMA, asymmetrical dimethylarginine; CLA, conjugated linoleic acid; SEM, standard error of the mean.

**Table 5:** Values of ΔADMA in the CLA and placebo groups

	Δ ADMA		
	
	CLA	Placebo	*p*
Median	0.04	−0.02	0.9618
First–third quartile	−0.14 to −0.13	−0.12 to −0.14	
Mean	0.01	−0.02	
SEM	0.06	0.06	

ΔADMA, change in asymmetrical dimethylarginine; CLA, conjugated linoleic acid; SEM, standard error of the mean.

## Discussion

Our study assessed the potential antiatherogenic effect of CLA supplementation in obese and overweight women. We studied the concentrations of selected markers of AT, hs-CRP and ADMA, that mainly reflect inflammatory status and endothelial function. They are also the most important markers of plaque formation, which is strongly connected with cardiovascular risk (12, 13). Our results do not show any impact of CLA supplementation on the aforementioned circulatory markers of AT.

The strengths of our study include its randomized, double-blind, placebo-controlled character, which reduced the possibility of bias and a relatively large percentage of participants who completed the study (85.14%). Furthermore, the study was limited to female participants to eliminate potential sex-related differences (14). The median age of study participants was below the traditionally established age of increased risk of endothelial dysfunction and cardiovascular disease (CVD) (55 years for females). Diabetes was also an exclusion criterion because of its high impact on the risk of atherogenesis (15). To avoid the impact of exercise on AT markers, the study participants were instructed to maintain their standard level of physical activity throughout the study. HT is an important risk factor of AT (15). Because of the common coexistence of HT and obesity the selection of a large group of obese subjects without HT was impossible. A limitation of the study is the small number of participants, according to a restriction of the sample size calculation described in our previous article (11).

Previous *in vitro* studies discovered several antiatherogenic properties of CLA (16) and demonstrated the cellular and molecular mechanisms of CLA anti-inflammatory activity (17). However, the results of human studies remain inconsistent. Reynolds and Roche (17) suggested the discrepancy between human and animal experiments may result from genetic variability in humans and larger amounts of CLA provided to animals with artificially induced, closely controlled disease.

The present study is the first study designed to assess the effect of CLA supplementation on selected AT markers in the population of obese and overweight females. Although there were several studies that reported a potential effect of CLA supplementation on CRP levels, they were performed with different CLA dosages, at different intervention times, or with different inclusion criteria. Furthermore, none of the previous studies assessed the effect of CLA supplementation on ADMA levels.

Sluijs et al. (18) reported no significant impact of CLA on CRP in overweight and obese adults. Although the aforementioned project was the most similar to our study, it was conducted using capsules with different c9t11CLA and t10, c12 CLA ratios (80% vs. 20%, respectively), that might have influenced the results, because these isomers have different effects. Moreover, the potential sex differences were not eliminated, because the study included both females and males (18). Raff et al. (19) investigated the effect of a CLA-rich fat diet on risk markers of AT, inflammation, type 2 diabetes, and lipid peroxidation in 38 healthy men. The results of this randomized, parallel intervention study are in agreement with our analyses; however, the Raff et al. study was conducted on a smaller group, used only young male subjects, and supplemented a higher CLA dosage. The study by Moloney et al. (20) focused on patients with type 2 diabetes. Plasma CRP concentrations were not altered by CLA or control supplementation; however, the data should be interpreted with caution because of the small sample size (16 participants in every group).

Steck et al. (21) reported that the consumption of 3 g/day of 50:50 mixture of c9t11 and t10c12 CLA for 12 weeks had no effect on the inflammatory markers of CVD that is consistent with our results. However, in the same study, when a higher CLA dosage was applied (6.4 g/day), CRP levels increased markedly (21). The results should be interpreted with caution because of the small number of participants in every studied group (*n=*18). Similarly, Smedman et al. (22) and Tholstrup et al. (23) showed the CRP level significantly increased after consumption of c9t11, t10c12 CLA mixture in high doses (4.2 g and 5.5 g, respectively).

There were only two studies performed with humans that demonstrated the decrease of CRP levels after CLA supplementation. Eftekhari et al. (24) studied the impact of CLA on inflammation in atherosclerotic patients. The 8-week-randomized clinical trial showed significant decreases in CRP concentration in the CLA group compared to the baseline, but this was not in comparison to the placebo group. Eftekhari et al. (24) suggested that the results were due to the low doses of t10,c12 CLA used in the trial, which has been reported as the isomer with several adverse effects on classical and novel markers of coronary vascular disease (2, 23). However, this hypothesis is not supported by the results of our study, because we used an identical dose and mixture ratio as Eftekhari et al. (24). Furthermore, they studied a group of non-obese volunteers with a history of angina, myocardial infarction, or bypass surgery. In Baghi et al. (25), the 2-week administration of CLA reduced serum concentrations of markers of inflammation (i.e. CRP, tumor necrosis factor-α) in healthy men after exhaustive exercise. However, their study is not comparable to our research because of a shorter time of CLA supplementation, different intervention characteristics (e.g. the participants underwent exhaustive exercise), and other inclusion criteria (e.g. young healthy males).

To our knowledge, this is the first study to assess the impact of CLA supplementation on ADMA levels, and it documents a lack of its action. ADMA is an endogenous inhibitor of endothelial nitric-oxide synthase. The reduction of nitric oxide results in endothelium dysfunction and local inflammation in consequence of activation of adhesion molecules and chemokines (26). Elevated plasma ADMA concentration has been identified as an independent risk factor for progression of AT and cardiovascular death (27). ADMA concentration is positively associated with low-density lipoprotein cholesterol concentration (28). Because CLA supplementation results in changes in the blood lipid profile (2, 29), we hypothesized that CLA may influence ADMA levels beneficially. However, we did not confirm this hypothesis.

The results of our research, as well as other clinical trials, do not confirm the positive results in animal studies. Therefore, further research on the effects and the safety of CLA supplementation should be conducted in large clinical trials.
